# Predictors of Pulmonary Hypertension and Right Ventricular Dysfunction in Patients with Hypersensitivity Pneumonitis

**DOI:** 10.3390/life13061348

**Published:** 2023-06-08

**Authors:** Natalia V. Trushenko, Olga A. Suvorova, Galina V. Nekludova, Iuliia A. Levina, Svetlana Y. Chikina, Alexandra M. Nikolenko, Natalia A. Tsareva, Alexandr V. Volkov, Andrey I. Yaroshetskiy, Zamira M. Merzhoeva, Galiya S. Nuralieva, Sergey N. Avdeev

**Affiliations:** 1 Pulmonology Department, Sechenov First Moscow State Medical University (Sechenov University), Healthcare Ministry of Russia, Trubetskaya St. 8, Build. 2, 119991 Moscow, Russia; olga.a.suvorova@mail.ru (O.A.S.); neklyudova_g_v@staff.sechenov.ru (G.V.N.); levina_yu_a@student.sechenov.ru (I.A.L.); chikina_s_yu@staff.sechenov.ru (S.Y.C.); nikolenko_a_m@student.sechenov.ru (A.M.N.); tsareva_n_a@staff.sechenov.ru (N.A.T.); sandyvlk@yahoo.com (A.V.V.); yaroshetskiy_a_i@staff.sechenov.ru (A.I.Y.); merzhoeva_z_m@staff.sechenov.ru (Z.M.M.); nuralieva_g@staff.sechenov.ru (G.S.N.); avdeev_s_n@staff.sechenov.ru (S.N.A.); 2 Pulmonology Scientific Research Institute, Federal Medical and Biological Agency of Russian Federation, Orekhovyy Boulevard 28, 115682 Moscow, Russia; 3 Federal State Budgetary Scientific Institution, V. A. Nasonova Research Institute of Rheumatology, Kashirskoye Highway 34A, 115522 Moscow, Russia

**Keywords:** hypersensitivity pneumonitis, pulmonary hypertension, right heart, pulmonary fibrosis, interstitial lung diseases, predictors

## Abstract

Background: Hypersensitivity pneumonitis (HP) is an interstitial lung disease (ILD) that occurs in susceptible individuals in response to various inhaled antigens. The fibrotic phenotype of HP is characterized by disease progression and can lead to pulmonary hypertension (PH). The aim of this study was to estimate the prevalence of PH and to identify predictors of PH in patients with chronic HP. Methods: We conducted an observational longitudinal study that included 85 patients with an established diagnosis of HP. Clinical examination, quality of life questionnaires, high-resolution computed tomography (HRCT) of the chest, arterial blood gases analyses, six-minute walking test (6-MWT), pulmonary function tests, and echocardiography were performed. Results: Patients were divided into groups with fibrotic (71.8%) and nonfibrotic phenotype (28.2%). PH was detected in 41 (48.2%) patients. Patients with PH had the predominant fibrotic phenotype of HP, were older, more symptomatic, and had a higher FVC/DLco ratio. The most significant predictors of PH were CT signs of fibrosis, finger clubbing, FVC/DLco, decreased distance, and SpO_2_ at the end of 6-MWT, as well as the presence of cardiovascular diseases. Conclusions: PH is a common condition in patients with chronic HP, especially with the fibrotic phenotype. Early detection of the PH predictors is necessary for the timely diagnosis of this complication of HP.

## 1. Introduction

Interstitial lung diseases (ILDs) comprise a heterogeneous group of diseases with variable pathophysiology, natural history, common functional characteristics (restrictive physiology and impaired gas exchange), and a common final pathway, eventually leading to irreversible fibrosis [1]. Therefore, findings in certain populations may not always be extrapolated to all types of ILDs [2].

It should be noted that in addition to the mechanisms of development of PH that are common for various ILDs—pulmonary fibrosis, comorbidities, hypoxemia, and inflammatory response, there are specific features for a particular disease.

Hypersensitivity pneumonitis (HP) is an immune-mediated ILD that manifests in susceptible individuals sensitized to inhaled antigens [3]. HP accounts for about 4–13% [4].

According to recent recommendations, HP is classified into nonfibrotic and fibrotic phenotypes based on the presence of fibrosis on histopathology and/or high-resolution computed tomography (HRCT) [3]. Development of pulmonary hypertension (PH) in the context of ILD is a well-recognized complication of various ILDs, including HP; however, the variable combination of lung parenchymal involvement and pulmonary vascular impairments in ILDs makes this patient population a difficult target for study [1,2]. Among patients with idiopathic pulmonary fibrosis (IPF) awaiting lung transplantation, most studies demonstrate a high prevalence of PH (39.7–84%) [5,6]. At the same time, PH can also be detected in patients with IPF without hypoxemia with mild to moderate impairment of lung function [7]. Data on PH prevalence among patients with HP are more limited; most authors report a frequency of 28.3–44.0% [8,9,10]. Several studies have confirmed a significant impact of PH on the risk of mortality in patients with IPF [7,11,12,13]. Hamada et al. showed that even a subclinical increase in mean pulmonary arterial pressure (mPAP) in patients with IPF awaiting lung transplantation is an important prognostic marker [14]. In patients with HP, the presence of PH was also associated with a fourfold increase in the risk of mortality [15]. The major problem of diagnosis PH in ILDs is non-specific clinical manifestations and limitations in instrumental diagnostic methods, including invasiveness and cost of right heart catheterization (RHC) and limited accuracy of transthoracic echocardiography (TTE). Therefore, identifying clinical markers of PH in patients with ILDs is an important task for the timely diagnosis of this condition. The aim of this study was to evaluate the frequency of PH and right ventricular (RV) dysfunction in patients with different phenotypes of HP and to identify predictors of PH development in patients with HP.

## 2. Materials and Methods

### 2.1. Study Design

This was an observational longitudinal study conducted in a pulmonology department of Sechenov University. The study was performed in accordance with the declaration of Helsinki and its subsequent revisions. Written informed consent was taken from all the patients.

### 2.2. Patients

The study included patients aged 18 years and older with HP, both fibrotic and nonfibrotic phenotypes. We used diagnostic criteria of HP according to the official 2020 ATS/JRS/ALAT Clinical Practice Guidelines [3]. All referred patients were reviewed by a multidisciplinary discussion team. A multidisciplinary diagnosis of HP was established based on the clinical history and/or HRCT patterns, plus the identification of plausible exposure and/or histopathological findings consistent with HP in lung biopsy samples and bronchoalveolar lavage fluid analysis. The fibrotic phenotype was determined by the predominant radiological and/or histopathological features of fibrosis. HRCT pattern of fibrotic HP required (a) HRCT pattern of lung fibrosis (irregular linear opacities/coarse reticulation with lung distortion; traction bronchiectasis and honeycombing) in one of the distributions (diffuse or in mild lung zone with relatively spared lower lung zones), and (b) at least one abnormality that was indicative of small airway disease (ill-defined, centrilobular nodules and/or ground-glass opacity with mosaic attenuation, three-density pattern, and/or air trapping) [3]. Typical histopathological features of fibrotic HP required chronic fibrosing interstitial pneumonia pattern (architectural distortion, fibroblast foci, subpleural honeycombing, fibrotic non-specific interstitial pneumonia-like pattern) or airway-centered fibrosis (peribronchiolar metaplasia, bridging fibrosis) and poorly formed non-necrotizing granulomas, cellular interstitial pneumonia, cellular bronchiolitis, organizing pneumonia pattern [3]. Nonfibrotic HRCT pattern required (a) at least one HRCT abnormality indicative of parenchymal infiltration (ground-glass opacity with mosaic attenuation) and (b) at least one HRCT abnormality indicative of small airway disease (ill-defined centrilobular nodules and/or air trapping) in diffuse distribution. Histopathological criteria for the diagnosis of nonfibrotic HP were cellular interstitial pneumonia, cellular bronchiolitis, and poorly formed non-necrotizing granulomas [3]. HRCT criteria for the usual interstitial pneumonia pattern were peripheral septal thickening, bronchiectasis, and honeycombing, mostly in the basal segments of the lungs.

### 2.3. Data Collection

The analysis included demographic and clinical characteristics: age, gender, duration of the disease, exposure, body mass index, smoking history, the modified Medical Research Council (mMRC) dyspnea score, the prevalence of comorbidities, the Charlson comorbidity index, the King‘s Brief Interstitial Lung Disease (K-BILD) score, European Quality of Life 5 Dimensions 5 Level Version (EQ-5D-5L) score, cough severity visual analog scale (VAS), presence of cyanosis, peripheral edema, and finger clubbing. We performed arterial blood gases analysis in room air at rest (GEM Premier 3500). A six-minute walk test (6-MWT) was performed according to the standard methodology [16]. Pulmonary function tests (spirometry, body plethysmography, and diffusive capacity of the lungs) were performed as routine measurements in all patients according to the ATS/ERS guidelines [17] and were reported as absolute values and percent of predicted values. Parameters were calculated according to the Global Lung Function Initiative [18]. Based on gender, age, forced vital capacity (FVC), and diffusing capacity for carbon monoxide (DLco) as a percent of predicted values, the Gender–Age–Physiology system (GAP) index was calculated. TTE was performed using Philips EPIQ 7 (Philips Healthcare) with a 1–5 MHz transducer. A transthoracic Doppler and two-dimensional images were obtained from parasternal long and short axes and apical four-chamber views. Tricuspid regurgitant flow was identified by color-flow Doppler techniques, and the maximum jet velocity was measured by continuous-wave Doppler. Echocardiographic parameters included in the analysis were linear dimensions of RV and left ventricular (LV), RV/LV dimension ratio, right atrium (RA) area, left atrium (LA) volume, the ratio of early (E) and late (A) transtricuspid inflow velocities (TV E/A ratio), systolic pulmonary artery pressure (sPAP), tricuspid annular plane systolic excursion (TAPSE), TAPSE/sPAP ratio, and ejection fraction. RV systolic pressure was estimated based on the modified Bernoulli equation and was considered equal to the sPAP in the absence of RV outflow obstruction [19]. TAPSE was assessed in the apical four-chamber view with the M-mode cursor through the lateral tricuspid annulus. TAPSE was measured as the peak excursion of the tricuspid annulus (millimeters) from the end of diastole to the end of systole, with values representing TAPSE being averaged over three to five beats. RV end-diastolic dimension was measured at the basal level of the RV cavity on the apical four-chamber view. RV end-diastolic dimension was measured at the basal level of the RV cavity on the apical four-chamber view. RV dilation was defined as RV end-diastolic dimension > 42 mm. RA dimension was calculated by the apical four-chamber view and was considered as increased if the area was >18 cm^2^ [20]. TAPSE/sPAP ratio < 0.55 mm/mm Hg has been proposed as a predictive risk factor for PH [21,22]. The medical records were carefully reviewed to exclude known causes of PH, including prior venous thromboembolism, LV dysfunction, human immunodeficiency virus infection, systemic vasculitis or other connective tissue diseases, chronic liver disease with portal hypertension, and a history of anorexigenic drugs uptake.

### 2.4. Statistical Analysis

Discrete variables were presented as frequencies and continuous variables as a median with an interquartile range (IQR). Continuous variables between groups were compared with the Mann–Whitney U-test. Categorical variables between groups were compared using Fisher’s exact test or Pearson’s chi-square test. We used logistic regression and multivariate regression analysis to assess the best echocardiographic predictors of PH. Odds ratios (OR) and 95% confidence intervals (CI) were calculated. Receiver operator characteristic curves (ROC) were used to calculate the sensitivity and specificity for PH predicting by risk factors and to determine the optimal prognostic cutoff values (Youden method). ROC analysis results were presented as an area under the curve (AUC), 95% CI, and diagnostic significance level (*p*). Differences were considered statistically significant at *p* < 0.05. Statistical data were processed using IBM SPSS Statistics software, version 26 (SPSS, Chicago, IL, USA).

## 3. Results

**Figure 1:**
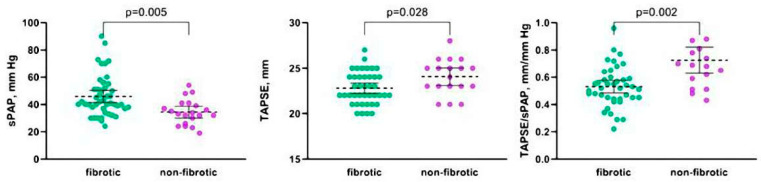
Comparison of sPAP, TAPSE, and TAPSE/sPAP in patients with fibrotic and nonfibrotic HP.

The study included 85 patients with HP: 24 patients with nonfibrotic phenotype and 61 patients with fibrotic HP phenotype; 31 patients (36.5%) had UIP-like patterns on HRCT. The baseline characteristics of patients are presented in Table 1. At the time of inclusion in the study, the presence of PH was recorded in 28 of 85 patients (32.9%), including 22 patients with fibrotic HP (36.1%) and 6 patients with nonfibrotic HP (25.0%). However, after performing echocardiography, PH was detected in 41 patients (48.2%), including 35 patients with fibrotic HP (57.4%) and 6 patients with the nonfibrotic phenotype (25.0%). A significant decrease in TAPSE (less than 17 mm) was not found in any patient. A decrease in TAPSE/sPAP below 0.55 was identified in 27 (31.8%) patients. Compared to patients with nonfibrotic HP, patients with fibrotic HP were older (65.0 (56.5–69.0) years vs. 49.0 (41.0–59.5) years; *p* = 0.0001), had greater mMRC dyspnea scale (3 (2–4) vs. 2 (1–3); *p* = 0.048), lower quality of life according to EQ-5D-5L questionnaire (self-related health 50 (30–70) vs. 75 (60–80); *p* = 0.001), higher cardiovascular comorbidity (45.9% vs. 16.7%, *p* = 0.007), higher GAP index (1 (1–2) vs. 1 (1–1); *p* = 0.003), shorter distance walked in 6-MWT (6-MWD) (332 (225–468) m vs. 443 (400–520) m; *p* = 0.006) and lower SpO_2_ at the end of 6-MWT (85 (79–88)% vs. 88 (84–92)%; *p* = 0.016). FVC and total lung capacity (TLC) did not differ significantly between the groups; however, DLco and FVC/DLco ratio differed in fibrotic HP vs. nonfibrotic HP (37.5 (29.0–55.0)% pred. vs. 55.5 (43.5–61.0)% pred.; *p* = 0.010, and 1.6 (1.3–2.1) vs. 1.3 (1.1–1.5); *p* = 0.005, respectively) (Table 1). A significant difference was found between fibrotic HP and nonfibrotic HP patients for the following echocardiography parameters: sPAP (40 (34–51) mm Hg vs. 33 (25–41) mm Hg, *p* = 0.005), TAPSE (23 (21–24) mm vs. 25 (23–26) mm, *p* = 0.028), TAPSE/sPAP (0.5 (0.5–0.6) vs. 0.7 (0.5–0.9), *p* = 0.002) and parameters of RV diastolic dysfunction (TV E/A ratio 0.7 (0.7–0.9) vs. 1.6 (1.0–1.6), *p* = 0.001), respectively (Figure 1). At the same time, there was no significant difference in the volume and pressure in LA volume and pressure, LV size, ejection fraction, and criteria of LV diastolic dysfunction (Table 1).

When dividing fibrotic HP patients into subgroups with UIP-like patterns and non-UIP-like patterns according to HRCT, a significant difference was found only in the length of the disease (24 (5–60) months vs. 4 (1–24) months, *p* = 0.02). No significant difference was revealed in the main clinical characteristics, lung function, and echocardiographic parameters.

**Figure 2:**
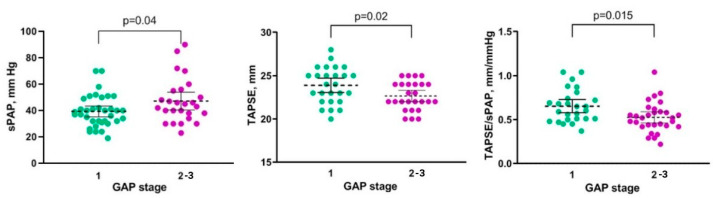
Comparison of sPAP, TAPSE, and TAPSE/sPAP in patients with 1 and 2–3 stages according to GAP.

When dividing all patients with HP in subgroups according to GAP stage, a significant difference was found in the sPAP (37 (31–42) vs. 44 (38–55), *p* = 0.040), TAPSE (24 (22–25) vs. 22 (22–24), *p* = 0.023), TAPSE/sPAP (0.63 (0.51–0.76)) vs. 0.50 (0.44–0.58), *p* = 0.015), TV E/A ratio (0.92 (0.80–1.45) vs. 0.77 (0.70–0.80), *p* = 0.020) for stages 1 and 2–3, respectively (Figure 2).

When dividing all patients with HP into subgroups with PaO_2_/FiO_2_ ≥ 300 mm Hg or <300 mm Hg, a significant difference was found only for the TAPSE (25 (23–25) vs. 22 (21–25), *p* = 0.030, respectively) and the TV E/A ratio (1 (0.78–1.15) vs. 0.67 (0.59–0.79), *p* = 0.050, respectively). There were no significant differences in sPAP, TAPSE/sPAP, or the size of the right heart chambers between these subgroups.

Compared to patients without PH, patients with PH were older (*p* = 0.006), had more prominent signs of fibrosis on HRCT (*p* = 0.002), had higher body mass index (*p* = 0.010), higher frequency of edema (*p* = 0.021) and finger clubbing (*p* = 0.031), more frequent history of cardiovascular diseases (*p* = 0.024), decreased physical tolerance (lower 6-MWD and lower SpO_2_ at the end of 6-MWT, *p* = 0.001 and *p* = 0.007, respectively), higher FVC/DLco ratio (*p* = 0.021). Patients with and without PH did not differ in other functional parameters. Additionally, there was no difference in pharmacological therapy for HP (Table 2).

When dividing patients with PH into subgroups with different stages by World Health Organization classification of functional status (WHO-FC), patients with III and IV classes were older (*p* = 0.049), had more severe GAP index (*p* = 0.039), more significant dyspnea according to mMRC scale (*p* = 0.013), higher frequency of cardiovascular diseases (*p* = 0.052), lower DLco % pred., and higher FVC/DLco ratio (*p* = 0.040 and *p* = 0.033, respectively) (Table 3).

According to the univariate regression analysis, the following factors predicted PH: HRCT signs of fibrosis (OR 5.5 (95% CI 1.7–17.6), *p* = 0.004) finger clubbing (OR 5.3 (95% CI 1.1–26.1), *p* = 0.040), FVC/DLco (OR 3.9 (95% CI 1.1–14.9), *p* = 0.040), cardiovascular comorbidity (OR 3.8 (95% CI 1.1–12.9), *p* = 0.030), SpO_2_ at the end of 6-MWT (OR 0.8 (95% CI 0.70–0.96), *p* = 0.010), 6-MWD (OR 0.99 (95% CI 0.98–0.99), *p* = 0.007), and age (OR 1.1 (95% CI 1.0–1.1), *p* = 0.005). The multivariate regression analysis showed that independent predictors of PH were HRCT signs of fibrosis (OR 6.4 (95% CI 1.6–24.7), *p* = 0.008) and cardiovascular comorbidity (OR 3.7 (95% CI 1.0–13.6), *p* = 0.050) (Table 4).

**Figure 3:**
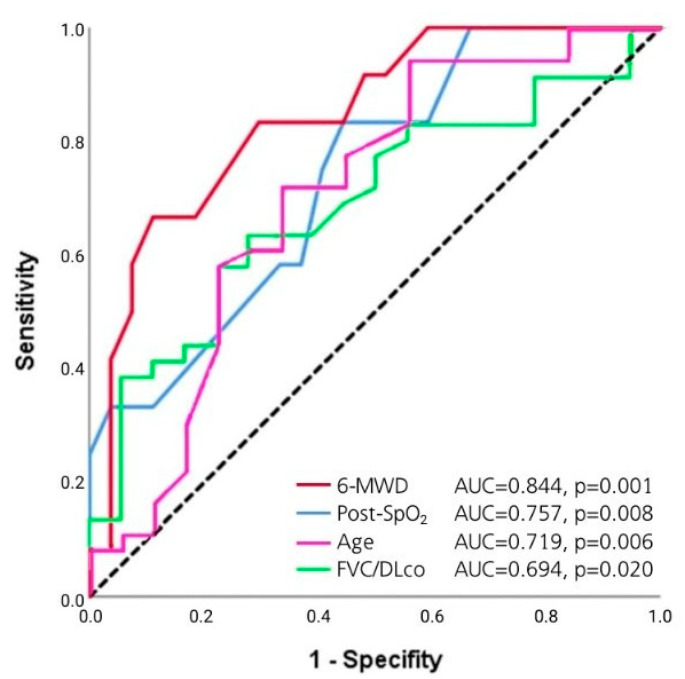
ROC-curves for PH and 6-MWD, SpO_2_ at the end of 6-MWT, age, FVC/DLco ratio.

ROC analysis showed that SpO_2_ at the end of 6-MWT can be used as a tool to predict PH in HP patients: SpO_2_ at the end of 6-MWT < 85% (Se 84.6%, Sp 55.2%, AUC 0.757 (95% CI 0.61–0.91; *p* = 0.008), 6-MWD < 390 m (Se 83.3%, Sp 70.4%, AUC 0.844 (95% CI 0.71–0.97); *p* = 0.001), FVC/DLco > 1.5 (Se 63.9%, Sp 72.2%, AUC 0.694 (95% CI 0.55–0.84), *p* = 0.020), age > 60 years (Se 65.0%, Sp 70.0%, AUC 0.719 (95% CI 0.58–0.86), *p* = 0.006) (Figure 3).

Among all patients included in the study, 27 patients (31.8%) had a TAPSE/sPAP ratio < 0.55. Patients with TAPSE/sPAP < 0.55 had longer disease duration (*p* = 0.013), more frequent fibrosis signs on HRCT (*p* = 0.016), cyanosis (*p* = 0.036), and finger clubbing (*p* = 0.010), as well as a history of cardiovascular diseases (*p* = 0.027), worse physical tolerance (lower 6-MWD and lower post-SpO_2_, *p* = 0.002 and *p* = 0.002, respectively), decreased PaO_2_ (*p* = 0.037) and DLco % pred. (*p* = 0.050) compared to those with TAPSE/sPAP ≥ 0.55 (Table 5).

The following factors predicted a significant decrease in TAPSE/sPAP: the finger clubbing (OR 5.3 (95% CI 1.4–19.9), *p* = 0.010), signs of fibrosis according to HRCT (OR 3.85 (95% CI 1.15–12.94), *p* = 0.028), history of cardiovascular diseases (OR 3.82 (95% CI 1.1–12.9), *p* = 0.030), SpO_2_ at the end of 6-MWT (OR 0.84 (95% CI 0.73–0.96), *p* = 0.008), DLco % pred. (0.96 (95% CI 0.93–0.99), *p* = 0.050), and 6-MWD (OR 0.99 (95% CI 0.985–0.996), *p* = 0.009).

According to the results of multivariate regression analysis, the most significant independent predictors of TAPSE/sPAP reduction were SpO_2_ at the end of 6-MWT (OR 0.83 (95% CI 0.72–0.95), *p* = 0.008) and cardiovascular comorbidity (OR 4.29 (95% CI 0.85–21.5), *p* = 0.078) (Table 6).

**Figure 4:**
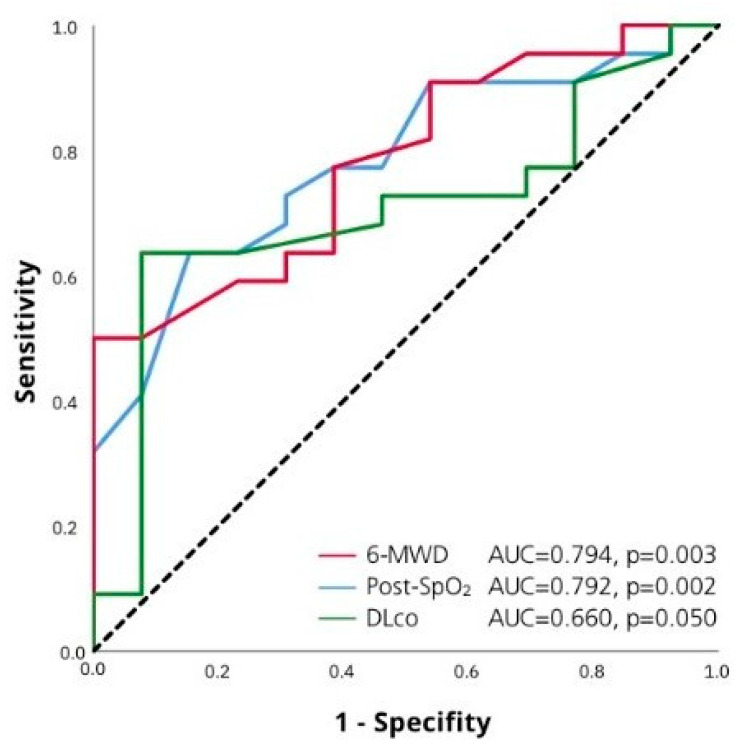
ROC-curve for TAPSE/sPAP less than 0.55 and 6-MWD, SpO_2_ at the end of 6-MWT, DLco % pred.

ROC-analysis revealed that independent predictors of TAPSE/sPAP reduction were SpO_2_ < 88% at the end of 6-MWT (Se 65.2%, Sp 84.8% (AUC 0.792 (95% CI 0.65–0.93), *p* = 0.002), 6-MWD < 410 m (Se 72.7%, Sp 64.3% AUC 0.794 (95% CI 0.69–0.94), *p* = 0.003), and DLco < 50% pred. (Se 60.7%, Sp 78.3%, AUC 0.660 (95% CI 0.51–0.81), *p* = 0.050) (Figure 4).

## 4. Discussion

In our study, PH was detected in 48.2% of patients, including 85.4% with a fibrotic phenotype. These results are comparable with the findings of Oliveira et al., who identified pre-capillary PH in 22 of 50 patients with chronic HP (44.0%) using RHC, with most patients having mPAP < 35 mm Hg [10]. Similar results were demonstrated by Dybowska et al., who detected PH in 26 of 70 patients with HP (37.0%) [8]. In a recent study by Elnady et al., the frequency of PH was slightly lower (28.3%) [9]. In another study, PH was detected in 19.0% of patients with HP using sPAP 50 mm Hg as TTE diagnostic threshold [23]. Therefore, different diagnostic methods and PH criteria, variability and inaccuracy of TTE results, as well as different disease severity and comorbidity in published studies on PH in patients with HP, should be taken into account. Variability in the frequency of PH in ILD patients depends on the period in the ILD course when PH is investigated [24], so we should mention that the median duration of HP was 10 (1–36) months in our study. Attention should be paid to a significant difference in the frequency of PH between medical documentation at the time of a patient’s inclusion in the study and the results obtained after the patient’s examination (TTE)—32.9% vs. 48.2%. The problem of underestimating PH in patients with HP has been confirmed by Wälscher et al., who assessed comorbidity in patients with chronic HP and showed a frequency of PH as high as 9.5% (20 out of 211 patients); this is significantly lower than the frequency of PH diagnosis based on medical records [8,10,15]. An important advantage of our study is an assessment of right heart size and function by TTE. According to the state-of-the-art, there is limited information on the prevalence of RV dysfunction and changes in the right heart size in patients with ILD, particularly with HP. According to our data, RA and RV dilation was detected in nine patients (10.6%), while all nine people had the fibrotic type of HP. TAPSE is the most commonly used and simple parameter for the evaluation of RV function, and depressed TAPSE portends a poor prognosis [25,26]. A decrease in TAPSE less than 16 mm is a predictor of the PH in patients with ILD (OR 7.80 955 CI (1.68–16.06), *p* = 0.009) [27]. We did not find a significant TAPSE decrease in our patients. Patients with PH significantly differed from patients without PH in the right heart size but not in TAPSE or TV E/A ratio. The interaction between preload, contractility, afterload, ventricular interdependence, and heart rhythm determines the regular RV function. In patients with HP, we found a predominance of pressure overload but not relevantly impaired RV contractility. IPF patients exhibited impairment of both systolic and diastolic RV functions, worse RV area change, greater RV dilation, and more RV free-wall hypertrophy compared to controls [28]. Alkukhun et al. reported that echocardiography showed mild to severe RV dysfunction in 22 (25.0%) IPF patients without PH and in 52 (54.0%) with PH (*p* < 0.001). TAPSE was decreased in IPF patients with and without PH (13 ± 4 vs. 14 ± 4, *p* = 0.31) [29]. Tornyos et al. showed that a combination of PH (increased sPAP > 35 mm Hg) and right heart strain occurred in 10.8% of patients with HP [30]. TAPSE/sPAP is a non-invasive, indirect measurement of RV contractile function and RV–pulmonary arterial coupling [31]. TAPSE/sPAP is a well-known potent independent predictor of pre-capillary PH and prognostic marker of all-cause mortality in PH, with a prognostic cutoff value of 0.55 mm/mm Hg and 0.36 mm/mm Hg, respectively [21,32,33,34,35]. According to our results, TAPSE/sPAP reduction < 0.55 mm/mm Hg was in 27 (31.8%) patients, and TAPSE/sPAP reduction < 0.36 mm/mm Hg was in only five patients (5.9%). RV diastolic dysfunction (TV E/A ratio < 0.8) was detected in 37.5% of patients, while the TV E/A ratio was significantly lower in patients with fibrotic HP. However, the influence of age on the TV E/A ratio [20] should be considered. Given the older age of patients with fibrotic HP, this could be significantly related to a lower TV E/A ratio. This marker of RV diastolic dysfunction may be clinically useful because it is an early and more easily quantifiable marker of subclinical RV dysfunction. Multiple studies demonstrated that RV diastolic dysfunction could be determined before apparent systolic dysfunction and RV dilation or hypertrophy appeared [20]. A significant difference was also obtained when comparing sPAP, TAPSE, and TAPSE/sPAP between fibrotic and nonfibrotic HP patients. The proportion of patients with PH in fibrotic HP was significantly higher (57.4 vs. 25.0%, *p* = 0.014). However, the UIP pattern in HP patients, according to our results, was not a significant predictor of PH. In a recently published study by Elnady et al., patients with HP with and without PH also differed in the predominance of fibrosis according to HRCT [9]. Similarly, Walscher et al. showed an association between the diagnosis of PH and the UIP pattern in patients with HP [15]. A number of experts suggest that extended fibrosis identified by chest HRCT and by histopathologic examination may be a significant etiological factor for PH development in advanced ILDs [11].

In addition to hypoxic vasoconstriction, a significant role in the pathogenesis of PH in fibrotic ILDs plays a complex interaction between epithelial cells, fibroblasts, and vascular cells, endothelial apoptosis, and growth factor-induced wall remodeling of the pulmonary artery, and destruction of the vascular bed and the lung parenchyma due to scar tissue occurrence [36]. At the same time, not all patients with pulmonary fibrosis develop PH, which indicates the heterogeneity of pathogenic mechanisms of PH in these patients [37]. Oliveira et al. did not reveal a relationship between histological patterns and PH in patients with HP [10]. The proportion of patients with fibrotic HP with and without PH did not differ significantly (66.0 vs. 61.0%, respectively); however, the authors noted that all patients with sPAP > 50 mm Hg had fibrotic HP [8].

The role of hypoxemia as a predictor of PH development in patients with ILDs was suggested in many studies. Elnady et al. showed that patients with HP and PH had significantly lower PaO_2_ (52.8 ± 10.4 vs. 75.3 ± 11.7, *p* < 0.001) [9] compared to those without PH. However, PH was also found in patients without hypoxemia. Therefore, PH in ILDs cannot always be explained by the presence of hypoxemia and the severity of pulmonary parenchyma damage; pulmonary vascular bed remodeling and other mechanisms may also play a role [1]. In our study, the only oxygenation parameter that differed significantly between patients with and without PH was SpO_2_ at the end of 6-MWT, which also was a significant predictor of PH in the regression analysis and was closely related to TAPSE/sPAP reduction. However, PaO_2_ was not a significant predictor of PH in our patients. Moreover, we did not find a significant difference in sPAP between patients with PaO_2_/FiO_2_ ≤ 300 mm Hg and >300 mm Hg, which could suggest a multifactorial nature of PH in HP.

According to our study, patients with and without PH differed in age, cardiovascular comorbidity, a number of clinical features, 6-MWT, and FVC/DLco. Interestingly, there were no significant differences in FVC, DLco, PaO_2_, mMRC, and quality of life. A significant difference was revealed for main PH-related parameters and the right heart function between subgroups with different GAP stages, which could confirm the importance of this index both for prognosis and for the comprehensive assessment of the risk of complications. The relationship between PH and lung function in patients with ILD is rather controversial [11,24]. According to Lettieri et al., a combination of decreased DLco less than 40% pred., need for supplemental oxygen, and decreased 6-MWT parameters predicted PH in patients with IPF [11]. This DLco threshold value for PH prediction in ILD patients was also confirmed by Parikh et al. [24]. Nadrous et al. demonstrated the strongest relationship between sPAP and DLco [13]. Oliveira et al. showed that reductions in FVC <60% pred. and in DLco < 50% pred. were significant predictors of PH in patients with HP, while FVC had a greater relationship with PH in the multivariate regression model [10]. However, Nathan et al. reported no significant correlations between FVC, Dlco, and the ratio of both to mPAP [38]. Koschel et al. did not show any difference in FVC % pred. and DLco % pred. between HP patients with and without PH [23]. The FVC/Dlco ratio was shown to be important in diagnosing PH in ILD patients [39,40,41]. Conceptually, greater values of this ratio may indicate a DLco that is low ‘out of proportion’ to the degree of interstitial fibrosis (estimated by FVC) [42]. Several different cutoff values for FVC/DLco were published, ranging from 1.4 to 2.2 [43].

In patients with systemic sclerosis, FVC/DLco ratio > 1.6 was suggested to be useful in predicting PH, regardless of the extent of pulmonary parenchymal involvement [39].

In IPF, a model using FVC/DLco along with baseline oxygen saturation was able to relatively accurately predict mPAP [42,44]. However, data regarding the role of the FVC/DLco ratio, including patients with HP, are controversial [8,45]. In our study, FVC/DLco was significantly higher in patients with PH, and its predictive value for PH was demonstrated in the univariate regression model but not in the multivariate regression analysis. We found a significant effect of PH on exercise tolerance in HP patients considering lower 6-MWD and lower SpO_2_ at the end of 6-MWT in PH patients. Desaturation at exertion could also be considered as a clinical marker of PH and decreased TAPSE/sPAP according to the regression analysis. SpO_2_ < 88% with a sensitivity of 65.2% and a specificity of 84.8% predicted TAPSE/sPAP decrease < 0.55 mm/mm Hg. Parikh et al. suggested that a distance < 350 m walked in 6-MWT could be one of the predictors of PH in ILD [24]. Patients with HP and PH also demonstrated worse results of 6-MWT, including shorter distance (441.2 vs. 511.9 m; *p* = 0.006) and more prominent desaturation (11.1 vs. 5.8%; *p* = 0.001). The authors proposed the following predictors of PH in patients with HP: 6MWD < 455 m and 6-MWT desaturation > 8% [8].

We also cannot exclude an influence of comorbidity, including cardiovascular disease, that is more frequent in fibrotic HP, although there were no significant differences in echocardiographic parameters related to the left heart function. Our study showed that patients with and without PH differed in the prevalence of cardiovascular diseases. Additionally, a history of cardiovascular diseases was related to the development of PH in the regression analysis. At the same time, one of the inclusion criteria in our study was the absence of LV diastolic or systolic dysfunction. Moreover, patients with and without PH did not differ in the main echocardiographic characteristics of the left heart. According to published data, LV dysfunction is not common in ILD patients without PH or cardiovascular comorbidities.

Accordingly, pulmonary capillary wedge pressure usually remains within the normal range [2].

We found that patients with HP have both similar and different predictors of PH with other ILD. Taken together, these observations contribute to the development of a strategy for monitoring patients with HP. Long-term prospective data will be necessary to confirm these findings.

## 5. Limitations

Our study had several limitations. Firstly, this was a single-center study with potential bias in patient selection. Our patient population was not skewed to those with more severe diseases, and a relatively high number of patients had well-preserved lung function and oxygenation status. Secondly, we did not use RHC and could not reliably exclude post-capillary PH in our patients. RHC is the “gold standard” for diagnosing PH in IPF. However, RHC is invasive, costly, and associated with complications. RHC is mostly carried out in a routine practice if future management would be influenced by RHC results, but there are no such data for PH in ILD. We assumed pre-capillary PH in these patients, but we cannot definitely exclude post-capillary PH in all of the patients. TTE is well-known to under- or over-estimate pulmonary pressure, especially in patients with ILDs, due to the limitations in the technique. TTE has limited accuracy in detecting early PH [37]. Overall, echocardiography proved itself as a tool to correctly predict PH with a sensitivity ranging from 0.79 to 1.00 and a specificity ranging from 0.60 to 0.98 [46]. However, despite these limitations, echocardiography is considered the best non-invasive method to screen for PH associated with lung diseases [47].

## 6. Conclusions

Overall, the prevalence of PH in patients with HP was 48.2%. CT signs of fibrosis, desaturation on exertion, FVC/DLco ratio, and the presence of cardiovascular diseases predicted PH in patients with HP. Our results also found a decrease in the TAPSE/sPAP and the TV E/A ratios in many HP patients with PH. Evaluation of the clinical role of these predictors requires further research in this area.

## Figures and Tables

**Table 1:** Comparison of patients with fibrotic and nonfibrotic phenotype of HP.

Parameters	Fibrotic HPMedian (IQR)/Frequency	Nonfibrotic HP Median (IQR)/Frequency	*p* Value
Age, years	65.0 (56.5–69.0)	49.0 (41.0–59.5)	0.0001
Disease duration, months	12.0 (2.0–36.5)	5.0 (0.0–36.0)	0.153
Gender (m/f), %	39.3/60.7	20.8/79.2	0.105
BMI, kg/m^2^	28.4 (24.3–32.3)	27.3 (22.7–29.2)	0.250
Smoking history, pack/years	0.0 (0.0–7.0	0.0 (0.0–10.5)	0.879
GAP stages	1 (1–2)	1 (1–1)	0.003
EQ-5D-5L:			
mobility	3 (2–4)	3 (2–3)	0.485
self-care	2 (1–4)	1 (1–2)	0.016
usual activities	3 (2–4)	2 (1–3)	0.027
pain/discomfort	2 (1–3)	2 (1–2)	0.363
anxiety/depression	2 (2–3)	2 (1–2)	0.043
self-rated health	50 (30–70)	75 (60–80)	0.001
K-BILD, points	54 (45–75)	61 (49–82)	0.188
mMRC, points	3 (2–4)	2 (1–3)	0.048
Cough, VAS, points	5 (2–7)	2 (1–7)	0.755
Cyanosis, %	11.5	4.2	0.322
Peripheral edema, %	11.5	4.2	0.322
Finger clubbing, %	29.5	12.5	0.099
Cardiovascular diseases, %	45.9%	16.7%	0.007
Charlson comorbidity index, points	4 (4–5)	3 (2–5)	0.012
6-min walking test:			
distance, m	332 (225–468)	443 (400–520)	0.006
pre-SpO_2_, %	94 (92–96)	95 (93–97)	0.239
post-SpO_2_, %	85 (79–88)	88 (84–92)	0.016
Hemoglobin, g/L	136 (126–145)	142 (130–152)	0.338
Erythrocytes, 10^12^/L	4.6 (4.2–5.2)	4.9 (4.5–5.2)	0.165
Leukocytes, 10^9^/L	8.1 (6.7–10.3)	7.4 (6.03–11.4)	0.421
Monocytes, 10^9^/L	0.6 (0.4–0.7)	0.5 (0.4–0.6)	0.931
Neutrophils to lymphocytes ratio	1.9 (1.4–2.5)	1.8 (1.4–2.5)	0.821
CRP, mg/L	6.1 (3.5–10.8)	5.9 (1.9–10.9)	0.650
FVC, % pred	65.5 (52.0–79.0)	68.5 (52.0–81.0)	0.862
FEV_1_, % pred	70.0 (55.0–84.5)	71.5 (57.0–83.5)	0.838
TLC, % pred	67.0 (51.0–77.0)	67.0 (58.0–85.0)	0.147
DLco, % pred	37.5 (29.0–55.0)	55.5 (43.5–61.0)	0.010
FVC/DLCO ratio	1.6 (1.6–2.1)	1.3 (1.1–1.5)	0.005
CPI	47.7 (39.7–58.9)	50.8 (36.9–61.2)	0.901
PaO_2_, mmHg	63.5 (58.8–72.8)	74.9 (58.8–86.3)	0.312
PaCO_2_, mmHg	37.9 (34.6–41.5)	36.5 (32.6–41.3)	0.381
RV basal diameter, mm	38.6 (35.5–39.4)	37.2 (35.6–38.5)	0.112
LV line size, mm	43.4 (43.0–47.1)	43.0 (43.0–46.4)	0.867
RV/LV	0.8 (0.8–0.9)	0.8 (0.8–0.9)	0.120
RA area, cm^2^	15.3 (13.4–18.5)	15.2 (13.1–16.5)	0.522
LA volume, mL	51.2 (45.8–57.3)	48.5 (44.5–54.5)	0.595
TV E/A ratio	0.7 (0.7–0.9)	1.6 (1.0–1.6)	0.001
Pulmonary artery diameter > 25 mm, %	11.5	0.0	0.052
Inferior vena cava diameter > 21 mm, %	1.6	12.5	0.020
sPAP, mmHg	40 (34–51)	33 (25–41)	0.005
TAPSE, mm	23 (21–24)	25 (23–26)	0.028
TAPSE/sPAP, mm/mm Hg	0.5 (0.5–0.6)	0.7 (0.5–0.9)	0.002
TAPSE/sPAP < 0.55, %	37.7	16.7	0.105
Pulmonary hypertension, %	57.4	25	0.014
EF, %	62 (61–63)	65 (63–67)	0.001

BMI, body mass index; GAP, the Gender–Age–Physiology system; EQ-5D-5L, European Quality of Life 5 Dimensions 5 Level Version score; KBILD, King’s Brief Interstitial Lung Disease questionnaire; mMRC, the modified Medical Research Council dyspnea score; 6-MWT, 6-min walking test; VAS, visual analog scale; CRP, C-reactive protein; FVC, forced vital capacity; FEV_1_, forced expiratory volume in 1 s; TLC, total lung capacity; DLco, diffusing capacity for carbon monoxide; CPI, composite physiological index; RV, right ventricle; RA, right atrium; LV, left ventricle; LA, left atrium; TV E/A, ratio of early (E) and late (A) transtricuspid inflow velocities; sPAP, systolic pulmonary artery pressure; TAPSE, tricuspid annular plane systolic excursion; EF, ejection fraction.

**Table 2:** Characteristics of patients according to the presence of pulmonary hypertension.

Parameters	PH Median (IQR)/Frequency	No PH Median (IQR)/Frequency	*p* Value
Fibrotic type, %	83.3	45.5	0.002
Age, years	66.5 (58.0–69.5)	56.0 (46.0–64.0)	0.006
Disease duration, months	10.0 (1.5–24.0)	6.0 (0.0–26.0)	0.227
Gender (m/f), %	40.0/60.0	33.3/ 66.7	0.610
BMI, kg/m^2^	29.2 (27.3–32.4)	26.2 (23.2–28.4)	0.010
Smoking history, pack/years	0.0 (0.0–7.5)	0.0 (0.0–13.0)	0.991
GAP, stage	2 (1–2)	1 (1–2)	0.235
EQ5D5L self-rated health, points	55 (35–73)	80 (50–80)	0.086
K-BILD, points	58 (50–75)	50 (46–86)	0.812
mMRC, points	3 (2–3)	3 (2–4)	0.776
Cough, VAS, points	5 (3–7)	2 (1–3)	0.075
Cough, %	84.2	60.0	0.041
Cyanosis, %	21.2	5.0	0.110
Peripheral edema, %	22.0	0	0.021
Finger clubbing, %	36.8	10.0	0.031
Cardiovascular diseases, %	61.8	30.0	0.024
Charlson comorbidity index, points	4 (4–5)	3 (2–5)	0.186
6-min walking test:			
distance, m	318 (220–400)	475 (400–520)	<0.001
pre-SpO_2_, %	94 (92–95)	95 (93–97)	0.135
post-SpO_2_, %	84 (78–88)	88 (86–93)	0.007
FVC, % pred	70.5 (55.8–79.5)	64.5 (48.0–77.5)	0.249
FEV_1_, % pred	72.5 (58.5–85.0)	62.5 (48.3–77.5)	0.196
TLC, % pred	67.0 (54.5–76.0)	65.0 (51.8–85.8)	0.854
DLco, % pred	38.3 (30.5–54.0)	50.2 (31.0–61.0)	0.363
FVC/DLco ratio	1.6 (1.3–2.2)	1.4 (1.3–1.5)	0.021
CPI	49.2 (38.8–60.9)	47/1 (37.6–60.2)	0.745
PaO_2_, mm Hg	62.0 (54.2–70.0)	70.0 (61.0–77.0)	0.220
PaCO_2_, mm Hg	35.0 (32.3–42.2)	36.0 (33.0–38.0)	0.919
RV basal diameter, mm	38.0 (36.0–39.0)	36.0 (35.0–38.0)	0.080
RA area, cm^2^	15.5 (14.2–18.0)	14.0 (11.8–15.6)	0.132
LV line size, mm	43.0 (43.0–47.5)	43.0 (42.3–43.0)	0.061
LA volume, mL	51.0 (45.0–57.0)	46.0 (44.0–54.0)	0.439
TV E/A ratio	0.77 (0.70–0.96)	1.30 (0.70–1.60)	0.209
sPAP, mm Hg	46 (40–54)	30 (26–32)	0.0001
TAPSE, mm	23 (21–25)	23 (22–25)	0.549
TAPSE/sPAP, mm/mm Hg	0.51 (0.44–0.56)	0.77 (0.70–0.88)	0.0001
TAPSE/sPAP <0.55, %	64.3	0.0	0.0001
UIP-pattern on HRCT, %	40.5	22.7	0.169
Long-term oxygen, %	28.6	10.5	0.128
Nintedanib treatment, %	11.8	5	0.408
SCS treatment, %	62.9	70	0.592
MMF treatment, %	5.7	15.0	0.249

PH, pulmonary hypertension; BMI, body mass index; GAP, the Gender–Age–Physiology system; EQ-5D-5L, European Quality of Life 5 Dimensions 5 Level Version score; KBILD, King’s Brief Interstitial Lung Disease questionnaire; mMRC, the modified Medical Research Council dyspnea score; 6-MWT, 6 min walking test; FVC, forced vital capacity; FEV_1_, forced expiratory volume in 1 s; TLC, total lung capacity; DLco, diffusing capacity for carbon monoxide; UIP-pattern, the usual interstitial pneumonia pattern; CPI, composite physiological index; RV, right ventricle; RA, right atrium; LV, left ventricle; LA, left atrium; TV E/A, ratio of early (E) and late (A) transtricuspid inflow velocities; sPAP, systolic pulmonary artery pressure; TAPSE, tricuspid annular plane systolic excursion; SCS, systemic corticosteroids; MMF, mycophenolate mofetil.

**Table 3:** Comparison of patients according to the World Health Organization classification of functional status of patients with pulmonary hypertension.

Parameters	WHO-FC I and II Median (IQR)/Frequency	WHO-FC III and IV Median (IQR)/Frequency	*p* Value
Fibrotic type, %	76.9	92.9	0.244
Age, years	60.0 (50.0–68.0)	67.5 (61.0–72.0)	0.049
Disease duration, months	6 (1–24)	11 (1–36)	0.639
Gender (m/f), %	46.2/53.8	35.7/64.3	0.581
BMI, kg/m^2^	28.6 (27.7–31.1)	28.9 (25.4–32.4)	0.923
Smoking history, pack/years	0.0 (0.0–20.0)	0.0 (0.0–10.0)	0.341
GAP, points	3 (2–4)	4 (3–5)	0.039
GAP, stage	1 (1–2)	2 (1–2)	0.060
EQ5D5L self-rated health, points	60 (50–70)	55 (25–77)	0.958
K-BILD, points	60 (50–79)	50 (42–75)	0.292
mMRC, points	2 (1–3)	3 (3–4)	0.013
Cough, VAS, points	5 (2–8)	5 (4–7)	0.882
Cough, %	53.8	71.4	0.345
Cyanosis, %	15.4	35.7	0.332
Peripheral edema, %	15.4	28.6	0.473
Finger clubbing, %	23.1	50.0	0.148
Cardiovascular diseases, %	30.8	71.4	0.052
Charlson comorbidity index, points	4 (2–5)	4 (4–6)	0.190
6-min walking test:			
distance, m	400 (345–448)	220 (115–285)	0.0001
pre-SpO_2_, %	94 (92–95)	95 (92–96)	0.244
post-SpO_2_, %	85 (77–88)	84 (80–88)	1.000
FVC, % pred	74.0 (54.0–80.0)	67.5 (56.8–75.8)	0.846
FEV_1_, % pred	73.0 (58.0–82.5)	75.0 (59.3–85.0)	0.716
TLC, % pred	68.0 (53.0–83.5)	61.0 (53.0–71.8)	0.423
DLco, % pred	41 (37–59)	33 (23–47)	0.040
FVC/DLco ratio	1.47 (1.20–1.80)	2.20 (1.62–2.27)	0.033
CPI	55.6 (37.1–63.7)	58.63 (41.5–61.7)	0.885
PaO_2_, mm Hg	64.0 (62.0–86.0)	44.0 (41.5–76.5)	0.153
PaCO_2_, mm Hg	33.4 (31.3–41.0)	34.5 (31.8–42.3)	0.816
UIP-pattern on HRCT, %	38.5	57.1	0.239
RV basal diameter, mm	38.0 (36.5–39.0)	37.0 (36.0–42.5)	0.856
RA area, cm^2^	16.0 (15.0–17.4)	15.7 (13.8–20.9)	0.616
LV line size, mm	44.0 (43.0–47.5)	43.0 (43.0–46.0)	0.616
LA volume, mL	50.0 (45.0–63.0)	48.0 (43.0–54.8)	0.268
TV E/A ratio	0.93 (0.72–1.00)	0.70 (0.64–0.92)	0.103
sPAP, mm Hg	41.0 (39.5–49.5)	46.0 (39.5–59.5)	0.253
TAPSE, mm	23 (22–25)	24 (22–25)	0.832
TAPSE/sPAP, mm/mm Hg	0.55 (0.51–0.59)	0.49 (0.40–0.57)	0.110
TAPSE/sPAP < 0.55, %	38.5	64.3	0.249

BMI, body mass index; GAP, the Gender–Age–Physiology system; EQ-5D-5L, European Quality of Life 5 Dimensions 5 Level Version score; KBILD, King’s Brief Interstitial Lung Disease questionnaire; mMRC, the modified Medical Research Council dyspnea score; 6-MWT, 6 min walking test; FVC, forced vital capacity; FEV_1_, forced expiratory volume in 1 s; TLC, total lung capacity; DLco, diffusing capacity for carbon monoxide; UIP-pattern, the usual interstitial pneumonia pattern; CPI, composite physiological index; RV, right ventricle; RA, right atrium; LV, left ventricle; LA, left atrium; TV E/A, ratio of early (E) and late (A) transtricuspid inflow velocities; sPAP, systolic pulmonary artery pressure; TAPSE, tricuspid annular plane systolic excursion.

**Table 4:** Univariate and multivariate regression analysis of the association between variables and development of PH.

Parameters	Univariate Analysis OR (95% CI)	Univariate Analysis *p*-Value	Multivariate Analysis OR (95% CI)	Multivariate Analysis *p*-Value
HRCT signs of fibrosis	5.5 (1.7–17.6)	0.004	6.4 (1.6–24.7)	0.008
Finger clubbing	5.3 (1.1–26.1)	0.040		
FVC/DLco ratio	3.9 (1.1–14.9)	0.040		
Cardiovascular diseases	3.8 (1.1–12.9)	0.030	3.7 (1.0–13.6)	0.050
Age	1.1 (1.0–1.1)	0.005		
Distance walked in 6-MWT	0.99 (0.98–0.99)	0.007		
Post-SpO_2_ 6-MWT	0.80 (0.70–0.96)	0.010		

FVC, forced vital capacity; 6-MWT, 6 min walking test; HRCT, high-resolution computed tomography.

**Table 5:** Characteristics of patients according to the presence or absence of TAPSE/sPAP < 0.55.

Parameters	TAPSE/sPAP ≥ 0.55 Median (IQR)/Frequency	TAPSE/sPAP < 0.55 Median (IQR)/Frequency	*p* Value
Fibrotic type, %	56.3	85.28	0.016
Age, years	66 (57–69)	60 (46–68)	0.177
Disease duration, months	1.5 (0.0–17.0)	23.0 (5.0–36.0)	0.013
Gender (m/f), %	37.5/62.5	40.7/59.3	0.799
BMI, kg/m^2^	29.2 (27.0–32.4)	28.1 (25.2–30.6)	0.278
Smoking history, pack/years	0.0 (0.0–13.0)	0.0 (0.0–10.0)	0.333
GAP, stage	2 (1–2)	1 (1–2)	0.129
EQ5D5L self-rated health, points	65 (30–80)	60 (40–75)	0.442
K-BILD, points	52.0 (46.5–82.0)	58.5 (49.5–73.5)	0.835
mMRC, points	2 (2–3)	3 (3–4)	0.073
Cough, VAS, points	2.5 (1.5–5.0)	5.0 (4.0–7.0)	0.114
Cough, %	70.6	73.3	0.863
Cyanosis, %	7.1	30.0	0.036
Peripheral edema, %	10.3	14.3	0.672
Finger clubbing, %	13.8	45.8	0.010
Cardiovascular diseases, 5	37.9	70.0	0.027
Charlson comorbidity index, points	4.0 (2.0–5.0)	4.0 (3.5–5.0)	0.531
6-MWT			
distance, m	418 (318–480)	270 (120–380)	0.002
pre-SpO_2_, %	95.0 (93.0–96.5)	94.0 (92.0–94.5)	0.191
post-SpO_2_, %	88.0 (84.5–90.5)	81.5 (77.0–86.0)	0.002
FVC, % pred	68.0 (55.0–79.0)	61.5 (54.5–74.0)	0.353
FEV_1_, % pred	72.0 (61.0–78.0)	67.7 (57.0–83.0)	0.809
TLC, % pred	67.0 (53.0–84.0)	64.0 (55.0–73.0)	0.268
DLco, % pred	52.5 (32.5–61.0)	37.0 (29.5–49.0)	0.050
FVC/DLco ratio	1.41 (1.31–1.78)	1.60 (1.29–2.21)	0.190
CPI	47.4 (39.1–63.7)	49.0 (38.6–58.8)	0.558
PaO_2_, mm Hg	70.0 (61.5–79.0)	62.0 (45.0–67.0)	0.037
PaCO_2_, mm Hg	35.4 (32.9–39.5)	37.5 (34.1–42.3)	0.377
UIP-pattern by HRCT,%	21.9	48.1	0.050
TV E/A ratio	0.89 (0.70–1.08)	0.73 (0.67–0.92)	0.374
sPAP, mm Hg	34 (30–39)	51 (45–60)	0.0001
TAPSE, mm	23 (22–25)	23 (21–25)	0.528
LV line size, mm	43.0 (43.0–47.5)	43.0 (43.0–46.0)	0.580
LA volume, mL	50.5 (45.0–55.5)	49.5 (45.0–58.8)	0.691
RV basal diameter, mm	37.0 (36.0–39.0)	38.0 (36.0–39.0)	0.580
RA area, cm^2^	15.3 (14.5–16.0)	16.5 (13.5–20.0)	0.544

PH, pulmonary hypertension; BMI, body mass index; GAP, the Gender–Age–Physiology system; EQ-5D-5L, European Quality of Life 5 Dimensions 5 Level Version score; KBILD, King’s Brief Interstitial Lung Disease questionnaire; mMRC, the modified Medical Research Council dyspnea score; 6-MWT, 6 min walking test; FVC, forced vital capacity; FEV_1_, forced expiratory volume in 1 s; TLC, total lung capacity; DLco, diffusing capacity for carbon monoxide; UIP-pattern, the usual interstitial pneumonia pattern; CPI, composite physiological index; RV, right ventricle; RA, right atrium; LV, left ventricle; LA, left atrium; TV E/A, ratio of early (E) and late (A) transtricuspid inflow velocities; sPAP, systolic pulmonary artery pressure; TAPSE, tricuspid annular plane systolic excursion.

**Table 6:** Univariate and multivariate regression analysis of the association between each variable and prediction of TAPSE/sPAP depression < 0.55.

Parameters	Univariate Analysis OR (95% CI)	Univariate Analysis *p*-Value	Multivariate Analysis OR (95% CI)	Multivariate Analysis *p*-Value
Finger clubbing	5.3 (1.4–19.9)	0.010		
HRCT signs of fibrosis	3.85 (1.15–12.9)	0.028		
Cardiovascular diseases	3.82 (1.1–12.9)	0.030	4.29 (0.85–21.5)	0.078
Post-SpO_2_ 6-MWT	0.84 (0.73–0.96)	0.008	0.83 (0.72–0.95)	0.008
DLco, % pred	0.96 (0.93–0.99	0.050		
Distance walked in 6-MWT	0.99 (0.98–0.99)	0.009		

DLco, diffusing capacity for carbon monoxide; 6-MWT, 6 min walking test; HRCT, high-resolution computed tomography.

## Data Availability

The dataset analysed during the current study is available from the corresponding author on reasonable request.
